# The effectiveness of surgical procedures to prevent post-hysterectomy pelvic organ prolapse: a systematic review of the literature

**DOI:** 10.1007/s00192-020-04572-2

**Published:** 2020-11-05

**Authors:** Greta Lisa Carlin, Barbara Bodner-Adler, Heinrich Husslein, Magdalena Ritter, Wolfgang Umek

**Affiliations:** 1 Department of General Gynecology and Gynecologic Oncology, Medical University of Vienna, Vienna, Austria; 2 Karl Landsteiner Institute of Special Gynecology and Obstetrics, Vienna, Austria; 3 Department of Gynecology and Obstetrics, Medical University of Innsbruck, Innsbruck, Austria

**Keywords:** Hysterectomy, Post-hysterectomy pelvic organ prolapse

## Abstract

**Introduction and hypothesis:**

Hysterectomy is one of the most commonly performed gynecological surgical procedures. One of the long-term risks associated with hysterectomy is the occurrence of pelvic organ prolapse (POP). To prevent post-hysterectomy POP, several suspension procedures are routinely performed at the time of hysterectomy. We performed a systematic review of published data in order to define the most effective surgical procedures for the prevention of post-hysterectomy POP.

**Methods:**

We performed a systematic review of the literature by searching PubMed, the Cochrane Library, EMBASE, Ovid MEDLINE, and clinicaltrials.gov up to 24 May 2020. The search strategy included the keywords hysterectomy, post-hysterectomy, prolapse, colposuspension, culdoplasty, McCall, and combinations thereof. The inclusion criterion was a surgical procedure at the time of hysterectomy to prevent de novo POP. The outcome was incidence of post-hysterectomy POP.

**Results:**

Six out of 553 retrieved studies met the methodological criteria for complete analysis. In this review, 719 women aged over 18 years were included. Only 2 studies were designed as prospective trials; however, only 1 compared women undergoing a procedure at the time of hysterectomy with controls. The prevalence of post-hysterectomy prolapse varied from 0% to 39%.

**Conclusion:**

A systematic review of published literature suggests that performing variations of McCall culdoplasty at the time of hysterectomy might be the most effective prophylactic surgical procedure for preventing post-hysterectomy pelvic organ prolapse.

**Electronic supplementary material:**

The online version of this article (10.1007/s00192-020-04572-2) contains supplementary material, which is available to authorized users

## Introduction

Hysterectomy is one of the most frequently performed gynecological procedures [1–5]. Although hysterectomy rates have started to decline in recent years [6–10], globally it remains the second most frequently performed gynecological operation, with more than 400,000 hysterectomies being performed in 2017 in the European Union alone [11]. Additionally, hysterectomy remains an important treatment option for a number of benign and malignant indications [3, 12–17]. Up to 90% of hysterectomies are performed to treat benign conditions such as dysfunctional uterine bleeding, dysmenorrhea, endometriosis, fibroids, or pelvic organ prolapse (POP) [15, 18, 19].

Like any major surgical procedure, hysterectomy puts patients at risk for a number of post-operative issues [20–23]. Pelvic organ prolapse as one of the long-term risks of hysterectomy is still under debate. Hendrix et al. performed a cross-sectional analysis and found no correlation between hysterectomy and the development of subsequent cystocele or rectocele in 10,727 women after hysterectomy compared with 16,616 women without hysterectomy [24]. However, several other studies have shown that hysterectomy independently increased the incidence of subsequent POP, especially when hysterectomy was performed for POP indication [25–31]. In fact, some studies even have shown that women after hysterectomy were 50% more likely to report symptoms of pelvic floor disorders [32].

Several suspension procedures at the time of hysterectomy have been suggested to prevent subsequent POP occurrence. McCall culdoplasty and sacrospinous ligament fixation have been suggested as preventive surgical procedures at the time of vaginal hysterectomy. Suturing the cardinal and uterosacral ligaments to the vaginal cuff has been suggested as a preventive procedure at the time of abdominal or laparoscopic hysterectomy [33, 34].

Although the American Association of Gynecologic Laparoscopists (AAGL) recommends apical fixation at the time of hysterectomy [35], recently, the rate of apical support procedures was reported to regress, from 4% of cases in 2004 to 2.5% in 2013 [36].

With regard to prolapse of the vaginal apex in particular, it has been hypothesized that the “disruption of the cardinal–uterosacral ligament complex” during hysterectomy decreases vaginal support [37]. Therefore, adequate support for the vaginal apex would be an essential component to reduce the incidence of post-hysterectomy apical POP [38, 39].

Few trials have compared the effectiveness of individual surgical procedures. The objective of this study was to perform a systematic review of surgical procedures at the time of hysterectomy to prevent any kind of post-hysterectomy prolapse.

## Materials and methods

**Fig. 1:**
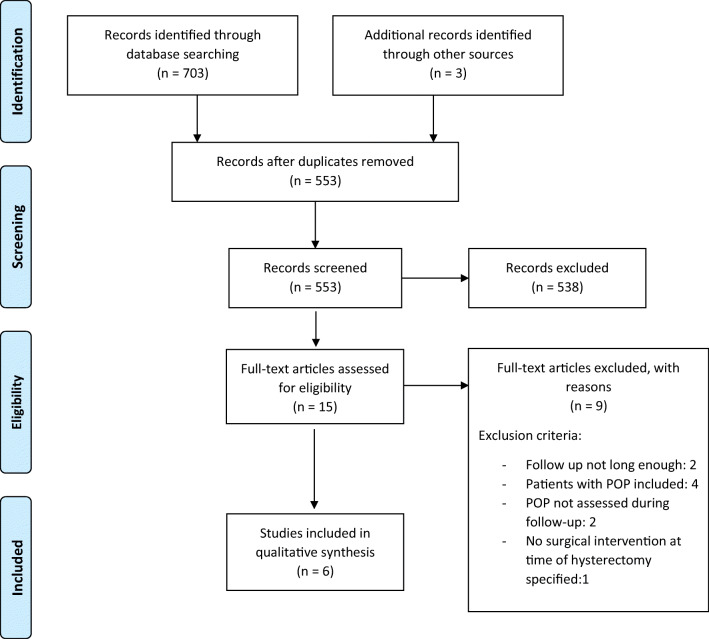
Preferred Reporting Items for Systematic Reviews and Meta-analyses flow diagram. *POP* pelvic organ prolapse

We performed a systematic review of the literature according to the guidelines of the Cochrane Handbook for Systematic Reviews of Interventions and the Preferred Reporting Items for Systematic Reviews and Meta-analyses (PRISMA). The databases consulted were PubMed, the Cochrane Library, EMBASE, Ovid MEDLINE, and clinicaltrials.gov up to 13 May 2020. This review was registered on PROSPERO (CRD42020148618). The protocol was initially proposed in April 2020. A search strategy was devised with the keywords “hysterectomy*,” “post-hysterectom*,” “prolapse*,” “colposusp*,” “culdoplast*,” “mccall*,” “mc call*,” and synonymous words or expressions. No bar on languages and no time limitation were set Fig. 1.

Inclusion criteria were meta-analysis, placebo-controlled randomized trials, systematic reviews, cohort studies, case series, and retrospective studies evaluating surgical procedures performed at the time of hysterectomy to prevent the occurrence of de novo post-hysterectomy pelvic organ prolapse (POP). The studies must investigate surgical procedures performed at the time of hysterectomy and describe the occurrence of post-hysterectomy POP as a main or secondary outcome. Subjects were women who underwent a hysterectomy for benign causes and had a follow-up at least 3 months after the surgery. The main outcome of our analysis was to detect the most effective surgical procedure to be performed at the time of hysterectomy in order to lower the incidence of de novo post-hysterectomy POP.

Exclusion criteria were studies that included women undergoing hysterectomy for malignancy or as a treatment of an existing POP. Studies that did not sufficiently describe the surgical procedure, or that had a follow-up period of less than 3 months, were also excluded. Data were categorized in terms of surgical approach to hysterectomy (vaginal, abdominal, laparoscopic) and type of surgical procedure performed at the time of hysterectomy to prevent post-hysterectomy POP.

The papers were selected by two of the authors (G.C., W.U.) of this study independently and then methodologically analyzed according to The Cochrane Risk of Bias tool for RCT (Table 1) and the Risk Of Bias In Non-randomized Studies—of Interventions (ROBINS-I) tool for assessment of methodological quality of non-RCTs (Table 2).

**Table 1:**
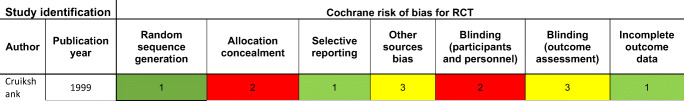
Cochrane risk of bias tool score for RCT

Each risk of bias answered by *Green Color 1* = “Low” (low risk of bias), *Red Color 2* = “High” (high risk of bias) or *Yellow Color 3* = “Unclear” (either lack of information or uncertainty over the potential for bias).

**Table 2:**
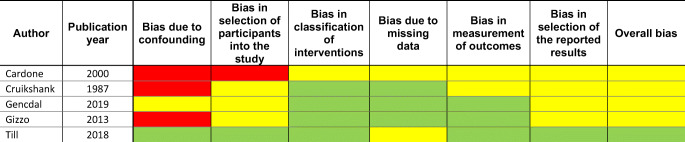
ROBINS-I tool for assessment of methodological quality of non-RCTs

*Green color* indicates low possibility of bias, *Yellow color* indicates moderate possibility of bias, *Red color* indicates serious possibility of bias.

## Results

Using our search strategy we identified 706 studies. This number includes studies extracted from each database along with related articles and references found in the selected studies. After deduplication, 553 studies remained. The abstracts of these 553 studies were read by two authors (G.C., W.U.) and 15 studies remained for a full analysis. Of these 15 studies 6 met the prerequisites for this systematic review (Table 3). The quality and heterogeneity of the studies included did not allow for an additional meta-analysis to be performed (Table 4). Of the 15 studies read in full, 8 studies were excluded as they did not report the incidence of post-hysterectomy POP, included women with POP at the time of hysterectomy, had too short a follow-up period, or all the above. One study was excluded because it did not study a specific surgical procedure to prevent subsequent prolapse, but compared supracervical hysterectomy plus abdominal sacral suspensions (ASC) with total abdominal hysterectomy (TAH) with ASC with regard to POP prevention [40]. We did not identify any previous systematic reviews or meta-analyses on this clinical question.

**Table 3:** Types of surgical fixation performed in the studies included

Intervention	Hysterectomy		
	Vaginal	Laparoscopic	Abdominal
McCall-type variations	[41]	[44, 45]	
Moschcowitz type	[41]		
Cruikshank method	[42]		
Fixation of USL* at the cervical portion	[43]		
Fixation of USL apex at the intermediate portion	[43]		
Uterosacral and cardinal ligament fixation			[46]
Uterosacral, cardinal, and round ligament fixation			[46]

*USL* uterosacral ligament

**Table 4:** Data extraction

Study identification									
Reference	Publication year	Study type	Type of hysterectomy performed	Suspension intervention performed	Control group, *n*	Study group, *n*	Follow-up period	Cases of POP at follow up, *n* (%)	*p* value
[46]	2000	Retrospective data analysis	Abdominal	Uterosacral and cardinal ligament fixation		133	1–12 years	20 (15)	No *p* value published
				Uterosacral, cardinal, and round ligament fixation		244		11 (4)	
[42]	1987	Prospective uncontrolled case series	Vaginal	Cruikshank method		112	7–42 months	0 (0)	No *p* value published
[41]	1999	Randomized controlled trial	Vaginal	McCall type	34	33	3 years	2 (6)	0.001
				Moschcowitz type		33		10 (30)	0.012
[43]	2013	Observational longitudinal cohort study	Vaginal	Fixation of uterosacral ligament stump at the intermediate portion	21	11	6 months	0 (0)	<0.01
				Fixation of uterosacral ligament stump at the cervical portion		10		4 (40)	
[44]	2019	Retrospective observational study	Laparoscopic	McCall type	20	18	2 years	Not published	0.03
[45]	2018	Prospective pilot study	Laparoscopic	McCall type	25	25	1 year	Not published	0.732

The six studies included in our review comprise a total of 719 women aged between 18 and 100 years and 10 different surgical procedures. Five of these 10 surgical procedures were performed as part of vaginal hysterectomy [41–43], 3 procedures were performed as part of laparoscopic hysterectomy [44, 45], and 2 procedures were performed as part of abdominal hysterectomy [46]. Two studies compared women undergoing a specific prophylactic surgical procedure with a control group of women without a prophylactic procedure during hysterectomy [41, 44]. Only 3 studies were designed as a prospective trial [41, 44, 45], of which only 1 included a control group with no suspension during hysterectomy [41]. Further, 1 study was an observational–longitudinal–cohort study [43], whereas the rest were retrospective data analyses.

Not all the surgical procedures performed at the time of hysterectomy to prevent post-hysterectomy POP were described in detail in every study. The procedures performed were sacral suspension, fixation of the vaginal apex to the uterosacral ligament, Moschcowitz-type fixation, and three different types of modified McCall culdoplasty (Table 3).

### Surgical procedures performed at the time of vaginal hysterectomy

#### McCall-type versus Moschcowitz-type versus peritoneal closure

Cruikshank and Kovac compared three different procedures at the time of vaginal hysterectomy to prevent post-hysterectomy enterocele in a case series of 100 women undergoing surgery for benign gynecological conditions. Four of these 100 women presented with POP [41].

In the first group, 33 women underwent a modified McCall culdoplasty, which elevated the posterior superior vaginal apex by suturing the uterosacral and cardinal ligaments of both sides to the peritoneum before being externalized through the vaginal wall.

In the second group, 33 patients underwent a modified Moschcowitz-type repair, which drew the supporting structures to the midline by suturing the uterosacral and cardinal ligaments of both sides to the vaginal apex. In addition, sutures connecting the peritoneum and the anterior wall of the rectum were applied before the sutures were passed through the peritoneum anteriorly. Thus, the supportive structures were drawn to the midline.

In the third group, 34 patients underwent peritoneal closing, wherein the peritoneum was sewn in a circumferential fashion around the vaginal apex opening. No attempt to tie the uterosacral and cardinal ligaments in the midline was made.

No prolapse was found at the 6-week and the 3-month follow-up. At the 3-year follow-up, 2 of the 32 women (6%) in the McCall culdoplasty group had developed a posterior–apical vaginal prolapse (stage 2), compared with 10 out of 33 women (30%) in the Moschcowitz-type group, and 13 out of 33 women (39%) in the peritoneal closure group. However, 4 of the 100 women included in the case series presented with POP at initial examination. Although the authors made no mention of these being symptomatic POPs or the surgical intervention taking place to correct the existing POP, bias cannot be ruled out. The authors concluded that compared with the Moschcowitz-type and peritoneal closure procedures, the McCall culdoplasty showed a statistically significant reduction of post-hysterectomy POP incidence (*p* = 0.004).

#### Cruikshank method

In another study, Cruikshank carried out a prospective uncontrolled case series performing vaginal hysterectomy for benign gynecological conditions in 112 consecutive patients [42]. To prevent subsequent vaginal vault prolapse and enterocele, the cardinal and uterosacral ligaments were sutured to the lateral angles of the vagina. Then, the peritoneum was closed, incorporating the cardinal and uterosacral ligaments, as well as the anterior rectal serosa, into the running purse string suture. Nineteen out of 112 patients initially presented with what the authors described as “symptomatic pelvic relaxation” and in these cases the cardinal and uterosacral ligaments were shortened in addition to being sutured to the vaginal apex.

During the follow-up period of 7 to 42 months, none of the patients presented with evidence for post-hysterectomy vaginal prolapse or enterocele. The author concluded that, although a longer follow-up period would be necessary, this additional procedure was promising in preventing the formation of enterocele after hysterectomy.

#### Fixation of the vaginal apex to the cervical versus the intermediate portion of the uterosacral ligaments

Gizzo et al. conducted a cohort study with 42 women undergoing hysterectomy either using LigaSure™ (group A, *n* = 21) or a clamp technique (group B, *n* = 21) [43].

Group A was further subdivided into subgroup A1, with 10 patients undergoing a suspension of the vagina to the cervical (more distal) portion of the uterosacral ligament, and subgroup A2, with 11 patients undergoing a suspension of the vagina to the intermediate (more cranial) portion of the uterosacral ligament. The description of the surgical procedure in group B mentions the “incorporation of the uterosacral–cardinal complex.”

After 6 months of follow-ups, 4 participants from subgroup A1 developed vaginal vault prolapse compared with none from subgroup A2, and none from group B. The authors attributed these prolapses to thermal damage of the uterosacral ligament and hypothesized that suspension of the vagina to the uterosacral ligament further away from the coagulation site might yield better results.

### Surgical procedures performed at the time of laparoscopic hysterectomy

#### McCall during laparoscopic hysterectomy

Gencdal et al. performed a retrospective observational study of 38 women, 18 of which had undergone laparoscopic hysterectomy with McCall culdoplasty (LH-McCall), and 20 had undergone laparoscopic hysterectomy without the McCall culdoplasty (LH) [44]. In the LH-McCall group, first the vaginal cuff was closed laparoscopically and the McCall culdoplasty was performed using a vaginal approach.

At the 2-year follow-up, apical support changes were more frequent in the control group than in the McCall group, with *p* = 0.03. However, the POP occurrence rates of each group are not included in the published paper. The authors concluded that the McCall culdoplasty was a safe and effective apical support to be performed at the time of hysterectomy.

#### Modified McCall culdoplasty

Till et al. performed a prospective pilot study of 50 patients undergoing laparoscopic hysterectomy, randomly assigning 25 patients to a two-layered cuff closure that incorporates the distal ends of the uterosacral ligaments by continuous suture and 25 patients to the same cuff closure with additional McCall culdoplasty [45]. In the baseline characteristics, the standard cuff closure group had significantly more relaxation of the anterior (POP-Q Aa −2.2 vs −2.6; *p* = 0.011) and posterior vaginal compartments (POP-Q Ba −2.2 vs −2.7; *p* = 0.006) preoperatively than the McCall culdoplasty group. The authors described none of these relaxations as symptomatic prolapse and the surgical procedures were not performed to treat existing POP; thus, the study was included in this review. However, bias cannot be ruled out.

At 12 months, 18 out of 25 patients in the McCall culdoplasty group (72%) and 16 out of 25 patients in the standard closure group (64%) returned for follow-up. No differences were found regarding apical descent during Valsalva or total vaginal length between groups. Also, there was no difference in operating time, estimated blood loss, surgical complications, or dyspareunia. The authors concluded that the addition of the McCall culdoplasty to total laparoscopic hysterectomy is feasible and safe and should therefore be considered in laparoscopic hysterectomy, as its prophylactic use to prevent future prolapse is well established in vaginal hysterectomy.

### Surgical procedures performed at the time of abdominal hysterectomy

#### Uterosacral and cardinal ligament fixation versus uterosacral, cardinal, and round ligament fixation

Cardone et al. conducted a retrospective analysis of 377 patients with hysterectomy for benign non-POP indications [46]. However, as 6 patients suffering from carcinoma in situ of the portio were included in the study group, there was an inconsistency in adhering to the inclusion criteria.

A total of 133 patients underwent fixation of the vaginal vault to the uterosacral and cardinal ligament, 244 patient underwent fixation of the vaginal wall to the uterosacral, cardinal, and round ligaments.

During the yearly follow-ups, starting after a minimum of 12 months and continuing up to a maximum of 12 years, 20 patients from the group with uterosacral and cardinal ligament fixation, and 11 patients from the group with additional round ligament fixation developed POP. The authors conclude that an additional fixation of the vaginal cuff to the round ligaments should be considered to further prevent post-hysterectomy prolapse. However, they cautioned against cuff fixation to the round ligament if excessive tension was necessary, as this has been described in previous literature to be painful for patients.

#### PULS study

We contacted the authors of the PULS trial, a randomized trial of Prophylactic Uterosacral Ligament Suspension at the Time of Hysterectomy for Prevention of Vaginal Vault Prolapse, but were unable to receive data for our review [47].

## Discussion

The findings of this systematic review suggest that performing McCall culdoplasty at the time of hysterectomy might reduce the risk of postoperative apical prolapse. The principle of the McCall culdoplasty is to elevate the vaginal vault and obliterate the posterior cul-de-sac. The strongest evidence for a decreased risk for the development of apical prolapse through the use of McCall culdoplasty compared with standard closure can be found for vaginal hysterectomy. This review also shows evidence for McCall-type procedure at the time of laparoscopic or abdominal hysterectomy to decrease the risk for the development of POP. The principle of McCall-type procedures is mainly to fix the vaginal apex to the uterosacral and cardinal ligaments without specifically obliterating the cul-de-sac.

Considering the anatomical proximity of the uterosacral ligaments to the ureters, there is a risk of ureteral injury when performing a McCall culdoplasty. However, early detection through cystoscopy at the time of hysterectomy reduces the chance of complications significantly [48]. None of the studies mentioned whether a routine cystoscopy was performed after hysterectomy to rule out bladder or ureteric injuries. Furthermore, McCall culdoplasty has been well established as a surgical treatment for post-hysterectomy POP, strengthening the point that McCall culdoplasty can counteract the native tissue support lost through hysterectomy [39, 49, 50].

Most of the studies included were found to be at risk of bias. The only RCT included in this review showed a high risk of bias in the allocation concealment and blinding. Of the 5 other studies included, 3 showed a high risk for bias due to confounding and 1 showed a high risk for bias in the participant selection. Often the risk of bias remained uncertain as the authors did not provide enough information in their published work for full assessment.

We detected a high degree of heterogeneity in the studies regarding the suspension procedures performed. Most authors performed modified McCall-type procedures, making comparison difficult. Furthermore, most studies did not provide detailed information on the fixation sites, i.e., where exactly on the ligaments the sutures were placed.

Additionally, there was no objective definition of prolapse that all the studies adhered to. The screening for prolapse was sometimes performed by measuring the total vaginal length or by subjective classifications, instead of using the standardized Pelvic Organ Prolapse Quantification System (POP-Q), making an objective comparison of the outcomes impossible. Till et al., for example, describe “preoperative relaxation” in the vaginal compartments of some participants, especially in the standard closure group, a fact that may have impacted the results [45]. Cruikshank et al. differentiated between “prolapse” and “pelvic tissue relaxation” and included patients with the latter in their studies, without giving further objective parameters that would allow for reproducibility [41, 42]. On the other hand, Cruikshank et al. published the only RCT and another trial with a long-term follow up period and well-described results. The data obtained from that study make a very strong point for the use of a suspension procedure at the time of hysterectomy to prevent subsequent prolapse.

Furthermore, we found the short duration of the follow-up period in most studies to be a major setback, as it is well established that the development of post-hysterectomy POP is a long-term risk.

The lack of RCTs and evidence we encountered on this subject is problematic. It has been well established that the risk of developing POP increases after hysterectomy [26] and around 200,000 women undergo pelvic floor surgical procedures in the USA alone [51] and that number is projected to further increase [32]. Additionally, the demand for health-care services related to pelvic floor disorders such as POP, is estimated to increase at twice the rate of the population itself, making the prevention of POP a pressing gynecological issue [52]. The strength of this study is the complete and systematic review of published data, including references, use of PRISMA criteria and including all languages to reach a conclusion.

We are aware of the shortcomings of this systematic literature review. Of the studies on the subject included, only 1 was designed as an RCT, whereas the rest either have no control group or are retrospective and observational in nature. Most studies conducted only a short-term follow-up, including only a small group size. In addition, patients suffering from different POP-Q stages pre-hysterectomy were often grouped together, making it difficult to obtain an objective analysis and interpretation of the data.

## Conclusion

Data on surgical procedures at the time of hysterectomy to prevent post-operative POP are scarce. Considering the large number of hysterectomies performed each year, the question of how to effectively prevent post-operative pelvic organ prolapse is important. Based on a systematic review of the current literature, we suggest performing a McCall-type procedure at the time of hysterectomy in order to prevent subsequent pelvic organ prolapse, but more well-conducted prospective studies are needed in order to solidify the evidence.

## Electronic supplementary material

ESM 1 (DOCX 20 kb)
