# Supplementary material for: The effectiveness of surgical procedures to prevent post-hysterectomy pelvic organ prolapse: a systematic review of the literature
Source: Int Urogynecol J. 2020 Nov 5;32(4):775–83. doi: 10.1007/s00192-020-04572-2 (PMC8009792; doi:10.1007/s00192-020-04572-2)
Supplement: Supplementary file 1 — (DOCX 20 kb) [file 192_2020_4572_MOESM1_ESM.docx]

Medline

| 1 | exp hysterectomy/ |
| --- | --- |
| 2 | hysterectom*.ti,ab,kf. |
| 3 | 1 or 2 |
| 4 | exp pelvic organ prolapse/ |
| 5 | prolapse*.ti,ab,kf. |
| 6 | 4 or 5 |
| 7 | 3 and 6 |
| 8 | (follow-up* or followup*).mp. |
| 9 | retrospectiv*.mp. |
| 10 | (month* adj3 (after or postoperat* or post-operat*)).mp. |
| 11 | (year* adj3 (after or postoperat* or post-operat*)).mp. |
| 12 | incidence*.mp. |
| 13 | prevalen*.mp. |
| 14 | cohort*.mp. |
| 15 | longitudinal*.mp. |
| 16 | (long-term or longterm).mp. or prevent*.ti. |
| 17 | 8 or 9 or 10 or 11 or 12 or 13 or 14 or 15 or 16 |
| 18 | 7 and 17 |
| 19 | study.mp,pt. |
| 20 | studies.mp,pt. |
| 21 | meta analysis.mp,pt. |
| 22 | systematic review.mp,pt. |
| 23 | randomized controlled trial*.mp,pt. |
| 24 | 19 or 20 or 21 or 22 or 23 |
| 25 | 18 and 24 |
| 26 | exp *hysterectomy/mt [methods] |
| 27 | exp *hysterectomy/ae [adverse effects] |
| 28 | post-hysterectom*.ti,ab,hw,kf. |
| 29 | posthysterectom*.ti,ab,hw,kf. |
| 30 | subsequen*.ti,ab,hw,kf. |
| 31 | suspen*.ti,ab,hw,kf. |
| 32 | colposuspen*.ti,ab,hw,kf. |
| 33 | (cuff adj4 clos*).ti,ab,hw,kf. |
| 34 | culdoplast*.ti,ab,hw,kf. |
| 35 | "mccall*".ti,ab,hw,kf. |
| 36 | "mc call*".ti,ab,hw,kf. |
| 37 | procedure*.ti. |
| 38 | technique*.ti. |
| 39 | method*.ti. |
| 40 | effect*.ti. |
| 41 | "after hysterectom*".ab. or (after and hysterectom*).ti. |
| 42 | (following and hysterectom*).ti. |
| 43 | (compara* or compare* or compari*).ti. |
| 44 | versus.ti. |
| 45 | vs*.ti. |
| 46 | 26 or 27 or 28 or 29 or 30 or 31 or 32 or 33 or 34 or 35 or 36 or 37 or 38 or 39 or 40 or 41 or 42 or 43 or 44 or 45 |
| 47 | 25 and 46 |

Embase

| 1 | 'hysterectomy'/exp |
| --- | --- |
| 2 | hysterectom*:ti,ab,kw |
| 3 | 1 OR 2 |
| 4 | 'pelvic organ prolapse'/exp |
| 5 | prolapse*:ti,ab,kw |
| 6 | 4 OR 5 |
| 7 | 3 AND 6 |
| 8 | 'follow up*' OR followup* |
| 9 | retrospectiv* |
| 10 | month* NEAR/3 (after OR postoperat* OR 'post operat*') |
| 11 | year* NEAR/3 (after OR postoperat* OR 'post operat*') |
| 12 | incidence* |
| 13 | prevalen* |
| 14 | cohort* |
| 15 | longitudinal* |
| 16 | 'long term' OR longterm OR prevent*:ti |
| 17 | 8 OR 9 OR 10 OR 11 OR 12 OR 13 OR 14 OR 15 OR 16 |
| 18 | 7 AND 17 |
| 19 | study |
| 20 | studies |
| 21 | 'meta analysis' |
| 22 | 'systematic review' |
| 23 | 'randomized controlled trial*' |
| 24 | 19 OR 20 OR 21 OR 22 OR 23 |
| 25 | 18 AND 24 |
| 26 | 'hysterectomy'/exp/mj AND 'surgical technique'/exp |
| 27 | 'risk factor'/exp AND 'postoperative complication'/exp |
| 28 | 'post hysterectom*':ti,ab,de,kw |
| 29 | posthysterectom*:ti,ab,de,kw |
| 30 | subsequen*:ti,ab,de,kw |
| 31 | suspen*:ti,ab,de,kw |
| 32 | colposuspen*:ti,ab,de,kw |
| 33 | (cuff NEAR/4 clos*):ti,ab,de,kw |
| 34 | culdoplast*:ti,ab,de,kw |
| 35 | mccall*:ti,ab,de,kw |
| 36 | 'mc call*':ti,ab,de,kw |
| 37 | procedure*:ti |
| 38 | technique*:ti |
| 39 | method*:ti |
| 40 | effect*:ti |
| 41 | 'after hysterectom*':ab OR (after:ti AND hysterectom*:ti) |
| 42 | following:ti AND hysterectom*:ti |
| 43 | compara*:ti OR compare*:ti OR compari*:ti |
| 44 | versus:ti |
| 45 | vs:ti |
| 46 | 26 OR 27 OR 28 OR 29 OR 30 OR 31 OR 32 OR 33 OR 34 OR 35 OR 36 OR 37 OR 38 OR 39 OR 40 OR 41 OR 42 OR 43 OR 44 OR 45 |
| 47 | 25 AND 46 |

Cochrane Central Register of Controlled Trials

| 1 | exp hysterectomy/ |
| --- | --- |
| 2 | hysterectom*.mp. |
| 3 | 1 or 2 |
| 4 | exp pelvic organ prolapse/ |
| 5 | prolapse*.mp. |
| 6 | 4 or 5 |
| 7 | 3 and 6 |
| 8 | (follow-up* or followup*).mp. |
| 9 | retrospectiv*.mp. |
| 10 | (month* adj3 (after or postoperat* or post-operat*)).mp. |
| 11 | (year* adj3 (after or postoperat* or post-operat*)).mp. |
| 12 | incidence*.mp. |
| 13 | prevalen*.mp. |
| 14 | cohort*.mp. |
| 15 | longitudinal*.mp. |
| 16 | (long-term or longterm).mp. or prevent*.ti. |
| 17 | 8 or 9 or 10 or 11 or 12 or 13 or 14 or 15 or 16 |
| 18 | 7 and 17 |
| 19 | exp *hysterectomy/mt [methods] |
| 20 | exp *hysterectomy/ae [adverse effects] |
| 21 | post-hysterectom*.mp. |
| 22 | posthysterectom*.mp. |
| 23 | subsequen*.mp. |
| 24 | suspen*.mp. |
| 25 | colposuspen*.mp. |
| 26 | (cuff adj4 clos*).mp. |
| 27 | culdoplast*.mp. |
| 28 | "mccall*".mp. |
| 29 | "mc call*".mp. |
| 30 | procedure*.ti. |
| 31 | technique*.ti. |
| 32 | method*.ti. |
| 33 | effect*.ti. |
| 34 | (after adj 2 hysterectom*).ab. or (after and hysterectom*).ti. |
| 35 | (following adj5 hysterectom*).ti. |
| 36 | (compara* or compare* or compari*).ti. |
| 37 | versus.ti. |
| 38 | vs*.ti. |
| 39 | 19 or 20 or 21 or 22 or 23 or 24 or 25 or 26 or 27 or 28 or 29 or 30 or 31 or 32 or 33 or 34 or 35 or 36 or 37 or 38 |
| 40 | 18 and 39 |

Cochrane Database of Systematic Reviews

| 1 | hysterectom*.ti,ot,ab,kw. |
| --- | --- |
| 2 | prolapse*.mp. |
| 3 | 1 and 2 |
